# Clinical utility of exome sequencing in individuals with large homozygous regions detected by chromosomal microarray analysis

**DOI:** 10.1186/s12881-018-0555-3

**Published:** 2018-03-20

**Authors:** Aparna Prasad, Matthew A. Sdano, Rena J. Vanzo, Patricia A. Mowery-Rushton, Moises A. Serrano, Charles H. Hensel, E. Robert Wassman

**Affiliations:** 1 Lineagen, Inc., 2677 East Parleys Way, Salt Lake City, UT 84109 USA; 2 Department of Biochemistry, University of Utah, Salt Lake City, USA

**Keywords:** Homozygosity, Exome sequencing, Diagnostics, Neurodevelopmental disorders, Pathogenic variants, Consanguineous, Consanguinity, Absence of heterozygosity, Long contiguous stretches of homozygosity, Runs of homozygosity, Clinical utility of genetic testing

## Abstract

**Background:**

Chromosomal microarray analysis (CMA) is recommended as the first-tier clinical diagnostic test for individuals with developmental disabilities. In addition to detecting copy number variations, CMA platforms with single nucleotide polymorphism probes can detect large homozygous regions within the genome, which represent potential risk for recessively inherited disorders.

**Methods:**

To determine the frequency in which pathogenic or likely pathogenic variants can be detected in these regions of homozygosity, we performed whole exome sequencing (WES) in 53 individuals where homozygosity was detected by CMA. These patients were referred to our clinical laboratory for a variety of neurodevelopmental conditions including autism spectrum disorder, developmental delay, epilepsy, intellectual disability and microcephaly.

**Results:**

In 11.3% (6/53) of cases, the analysis of homozygous variants revealed pathogenic or likely pathogenic variants in *GJB2*, *TPP1*, *SLC25A15*, *TYR*, *PCCB*, and *NDUFV2* which are implicated in a variety of diseases. The evaluation of heterozygous variants with autosomal dominant inheritance, compound heterozygotes and variants with X-linked inheritance revealed pathogenic or likely pathogenic variants in *PNPLA4*, *CADM1*, *HBB*, *SOS1*, *SFTPC*, *OTC* and *ASMT* in 15.1% (8/53) of cases. Two of these patients harbored both homozygous and heterozygous variants relevant to their phenotypes (*TPP1* and *OTC*; *GJB2* and *ASMT*).

**Conclusions:**

Our study highlights the clinical utility of WES in individuals whose CMA uncovers homozygosity. Importantly, we show that when the phenotype is complex and homozygosity levels are high, WES can identify a significant number of relevant variants that explain neurodevelopmental phenotypes, and these mutations may lie outside of the regions of homozygosity, suggesting that the appropriate follow up test is WES rather than targeted sequencing.

**Electronic supplementary material:**

The online version of this article (10.1186/s12881-018-0555-3) contains supplementary material, which is available to authorized users.

## Background

Neurodevelopmental disorders (NDDs) are characterized by brain dysfunction, which results in the impairment of one or more features including motor skills, learning, cognition, speech and language and personal or social development. NDDs include developmental delay (DD), autism spectrum disorder (ASD), intellectual disability (ID), learning disability, attention-deficit hyperactivity disorder (ADHD) and others. Genetic causes contribute significantly to the etiology of these disorders, and in recognition of this, the American College of Medical Genetics and Genomics (ACMG) has published practice guidelines recommending CMA as the first-tier clinical diagnostic test for individuals with certain NDDs and congenital anomalies [1]. Many genomic microarray platforms in use in clinics today employ a combination of probes to detect copy number variants (CNVs) as well as to genotype single nucleotide polymorphisms (SNPs). In addition to detecting copy number gains and losses, these microarray platforms can thus detect regions of homozygosity throughout the genome. It is hence not uncommon for diagnostic laboratories to identify patients with long contiguous stretches of homozygosity during routine CMA testing. These stretches of homozygosity are often called ‘runs of homozygosity’ (ROH) or regions with ‘absence of heterozygosity’. ROH may be caused by uniparental isodisomy or identity by descent [2, 3], and may be suggestive of an autosomal recessive cause for a patient’s condition. In such cases, WES is likely to provide the most comprehensive and efficient means of identifying causal genetic variants.

In this study, we performed whole exome sequencing (WES) on 53 patients with ROH that were identified by CMA, with the goal of determining the diagnostic utility of WES in these patients. These individuals were referred to our laboratory for clinical CMA testing for a variety of clinical features including ASD, DD, epilepsy, anxiety, ID, microcephaly and encephalopathy. We investigated variants throughout the exome and identified homozygous pathogenic or likely pathogenic variants that explain at least part of the indicated phenotype in 11% (6/53) of cases. An additional analysis of heterozygous mutations with autosomal dominant inheritance as well as X-linked variants identified pathogenic/likely pathogenic variants in 15% (8/53) cases. Two of these cases (ROH44 and ROH52) harbored both homozygous and heterozygous pathogenic/likely pathogenic variants. This study strongly supports the use of WES in individuals with ROH detected by CMA and further highlights the clinical utility of WES in these individuals.

## Methods

### Patient samples

All patient DNA samples were collected as part of standard clinical genetic testing by Lineagen, Inc. We obtained a waiver of consent from Western Institutional Review Board (IRB) Protocol No. 20162032, for this retrospective study. All patient data submitted to us for the purposes of clinical testing are covered under this IRB. Data was analyzed in a coded fashion in compliance with our IRB protocol. DNA isolated from buccal swabs (DNA Genotek, OC-100) using the PureGene extraction kit (Qiagen, Inc.) was used for all genetic analyses described in this study.

### Microarray analysis

Clinical CMA was performed using an optimized, ultra-high-resolution custom Affymetrix microarray that includes all functional content present on the Affymetrix CytoScan-HD microarray with probes added to improve detection of copy number variants associated with pediatric neurodevelopmental disorders [4]. DNA samples (250 ng) were digested, processed, labeled and hybridized to microarrays using the manufacturer’s recommended protocol (Affymetrix, Inc., Santa Clara, CA). Array results were interpreted by ABMGG-certified clinical cytogeneticists using the Chromosome Analysis Suite v2.0.1 software (Affymetrix, Inc., Santa Clara, CA). To estimate the percentage of genome homozygosity, all ROH regions greater than 3 Mb are totaled for the autosomes (X chromosome excluded to avoid male/female bias) and the total is divided by the total size of the autosomes. Samples were randomly selected for exome sequencing if the report of the clinical cytogeneticist stated ROH to be a potential concern. ROH were considered as a potential concern by the clinical cytogeneticist if the total ROH encompasses greater than 3% of the genome. Additionally, for cases with ROH between 1 and 3%, the cytogeneticist compared the ROH of the patient with common ROH in control populations and decides to flag these as concerning if there are significant disparities.

### Exome sequencing

Exome capture was performed using the Life Technologies AmpliSeq Exome RDY kit (Thermo Fisher, Carlsbad, CA) and sequencing was performed on Life Technologies Ion Proton sequencers with 200 bp reads using a PIV3 chip. Whole exome sequencing for all the patients was performed on a research basis. Sequences were aligned with and compared to the Genome Reference Consortium Human Build 37 (GRCh37, hg19) using the Ion Torrent Suite software v4.2 (ThermoFisher Scientific).

### Analysis of sequence data

Sequence data were analyzed using the default settings of the Clinical Sequence Analyzer (CSA) tool from WuXi NextCODE (https://www.wuxinextcode.com/). Homozygous and heterozygous likely protein-altering variants (nonsense, missense and splice sites) were analyzed and were assessed for predicted deleterious effects by Variant Effect Predictor (VEP) (score ≥ 0.9) from WuXi NextCODE [5]. VEP uses information from SIFT (Sorting Intolerant From Tolerant) [6] and PolyPhen-2 (Polymorphism Phenotype) [7, 8] to predict the impact of the variants on genes, transcripts and protein sequences. Insertion/deletion variants were removed from the analysis due to a high rate of false positive insertion/deletion variants in this data set. We acknowledge that some true positive variants may have been excluded because of this approach. Variants with allele frequencies > 0.001 in the Exome Aggregation Consortium [9] were removed as this allele frequency cutoff was demonstrated to be a justifiable threshold for a variety of human disease phenotypes without discarding true pathogenic variants [10]. WuXi NextCODE flags a variant as pathogenic or likely pathogenic if the variant has been reported as pathogenic in a previous peer reviewed publication or in public databases such as HGMD, ClinVar or OMIM. Variants within two base pairs of these pathogenic variants intended to capture variants within the same codon were also analyzed. Alignments for the final variants were manually inspected to verify read quality. The variants relevant to the phenotype of the patients were classified according to the ACMG standards and guidelines [11]. A detailed workflow showing the variants discovery and prioritization has been shown in Additional file 1.

## Results

### Genetic architecture of the patient cohort

We report here the results of whole exome sequencing in 53 individuals with ROH detected by clinical CMA. Our cohort comprised 33 males (62%) and 20 females (38%) with ages ranging from 6 months to 22 years. The male-to-female ratio in this cohort is similar to that of our overall clinical testing population. The percentage of the genome that is homozygous in this cohort varied from 1.4% to 25%. The patient (ROH12) with a ROH of 1.4% of the genome has a large ROH of 26 Mb on chromosome 3 and it may harbor genes for autosomal recessive disorders. This region of 26 Mb is not a common ROH in normal control populations and thus was included in this cohort. In total 16/53 of our patients have 1.4–3.0% ROH, 28/53 of patients have 3–10% ROH, 7/53 of patients have 10–20% ROH, and 2/53 of patients have 20–25% ROH. Two patients were identified by CMA (ROH36 and ROH38) with ROH in a single chromosome, 6 (3.49% ROH) and 16 (1.53% ROH) respectively, suggesting uniparental isodisomy. In the remaining patients, we identified long contiguous stretches of homozygosity on multiple chromosomes. In addition to ROH, 3/53 patients had pathogenic CNVs: patient ROH40 had a gain of the entire Y chromosome (47,XYY syndrome), patient ROH34 had a 2.54 Mb 22q11.21 deletion (DiGeorge syndrome) and patient ROH39 had a 637 kb 16p12.2 deletion. In 40/53 patients, no clinically reportable CNVs were identified and in 10/53 patients, a CNV of uncertain clinical significance was detected using CMA. Detailed phenotypic information including percentage of the genome that in homozygous in these patients as well as the CMA findings are provided in Additional file 2.

### Homozygous mutations

#### Known pathogenic or likely pathogenic variants

We analyzed homozygous, protein-disrupting variants and as expected we observed a strong linear correlation (Correlation coefficient of 0.85) between the percent of the genome with homozygosity and the number of predicted or known likely protein disrupting homozygous variants (Additional file 3). We found variants that met ACMG standards and guidelines for pathogenic or likely pathogenic status [11] in 6/53 (11%) cases. All of these variants were located within regions of homozygosity detected using chromosomal microarray analysis. The genes in which these variants were found encode a functionally diverse set of proteins involved in a number of different disorders relevant to the patients’ clinical features (Table 1). As examples, we present the potential impact of genetic diagnosis in three of these cases below.

**Table 1:** Details of homozygous pathogenic or likely pathogenic variants discovered in this study

Sample	% ROH	Gene	Chr	Position	HGVS (nucleotide)	HGVS (protein)	Variation type	ExAC Allele Count/Total Allele No.*	Classification	Associated Disorders
ROH04	13.5	*TYR*	11	88,924,546	c.996G > A	p.M332I	Missense	0/121412	Pathogenic	Oculocutaneous albinism type 1
ROH22	8.9	*PCCB*	3	136,002,730	c.346C > T	p.P116S	Missense	124/121382	Likely pathogenic	Propionic acidemia
ROH26	4.34	*SLC25A15*	13	41,381,541	c.564C > G	p.F188L	Missense	1/121412	Likely pathogenic	hyperornithinemia-hyperammonemia-homocitrullinuria (HHH) syndrome
ROH31	5.0	*NDUFV2*	18	9,117,901	c.120 + 5_120 + 8delGTAA	–	Splice Donor	0/121412	Pathogenic	Mitochondrial complex 1 deficiency
ROH44	24.55	*TPP1*	11	6,638,271	c.622C > T	p.R208*	Stop-gain	21/121276	Pathogenic	Neuronal ceroid lipofuscinosis
ROH52	18.1	*GJB2*	13	20,763,452	c.269 T > C	p.L90P	Missense	107/121346	Pathogenic	Deafness

*For all these variants, there were no homozygotes reported in ExAC

### *TPP1-*pathogenic variant

Patient ROH44 was referred to our laboratory with indications of encephalopathy, severe DD, lack of speech, frequent brief absences and drop spells, seizures and intellectual disability. Parents reported themselves as first cousins, and 25% of the autosomal genome was found to be homozygous using CMA. No clinically relevant CNVs were present. Using WES, we identified a homozygous known pathogenic stop-gain mutation (c.622C > T; p.R208*) in *TPP1*, also known as *CLN2* [12]. Mutations in *TPP1* cause an autosomal recessive neurodegenerative lysosomal storage disorder known as neuronal ceroid-lipofuscinosis (NCL). Late-infantile NCL (LINCL), also known as Jansky-Bielschowsky disease, typically presents between two and four years of age with seizures, and clinical features progress as the patient ages. In a study of 74 patients with LINCL, the p.R208* variant observed in our patient accounted for ~ 28% of the mutant alleles in *TPP1*, including five samples that were homozygous for this variant [12]. A diagnosis of LINCL is consistent with our patient’s age, phenotype, and genotype and establishes an expected disease progression. This diagnosis is critical to patient management and may provide a treatment approach given a recently completed clinical trial for provision of recombinant human tripeptidyl peptidase-1 therapy (ClinicalTrials.gov Identifier: NCT01907087).

### *SLC25A15*-likely pathogenic variant

Patient ROH26 was referred for CMA testing due to delayed milestones, speech and language deficits, and autism spectrum disorder (ASD). There was not a prior report of parental relatedness, and 4.34% ROH as well as a 9p24.3 duplication classified as a variant of uncertain clinical significance were identified on CMA. Using WES, we identified a homozygous variant (c.C564G; p.F188L) in *SLC25A15*, the gene encoding the mitochondrial ornithine transporter. This variant has been classified as pathogenic for hyperornithinemia-hyperammonemia-homocitrullinuria (HHH) syndrome [13]. HHH syndrome is an autosomal recessive disorder that can present as an acute or chronic disorder. It is highly heterogeneous, ranging from a mild form with slight intellectual disability and neurological involvement to a severe form with coma, lethargy, hepatic signs, and seizures [14]. Because elevated plasma ornithine levels may not manifest in the first days of life, HHH syndrome is often missed in newborn screening panels [15]. The p.F188 L variant found in our patient has been seen in another patient who presented with intellectual disability and mild liver disease at the age of 3.8 years [13]. Our patient presented at a similar age with delayed milestones, speech and language deficits, and ASD. These features are consistent with a chronic form of relatively mild HHH syndrome. A diagnosis of HHH syndrome can be confirmed through metabolic screens including plasma ammonia, plasma amino acid, urine amino acid, urine organic acid, and urine orotic acid analyses, and dietary intervention may help with some of the observed symptoms [16].

### *NDUFV2*-pathogenic variant

Patient ROH31 was referred to our laboratory for clinical testing based on delayed milestones and hypotonia. Parents reported themselves as second cousins. No clinically relevant CNVs were identified by CMA, but 5% of the autosomal genome was homozygous. Using WES, we identified a homozygous four base pair deletion in a splice site in intron 2 (c.120 + 5_120 + 8delGTAA) of *NDUFV2*, the gene encoding the NADH: ubiquinone oxidoreductase subunit of the inner mitochondrial membrane complex. This mutation was observed previously in three siblings of a consanguineous family who presented in the first year of life with hypertrophic cardiomyopathy, truncal hypotonia, feeding difficulties, and growth delay [17]. Functional studies performed by these authors revealed that the mutation led to skipping of exon 2, which contains the mitochondrial localization signal, and, consequently, reduced mitochondrial NDUFV2 function. Considering the young age coupled with generic and emerging symptoms of delayed milestones and hypotonia in this patient, it is likely that the *NDUFV2* variant is causative for this patient’s features. Since this mutation in *NDUFV2* is known to cause hypertrophic cardiomyopathy [17], monitoring for this type of defect in heart muscle would be included as a medical management change. Further, there is some suggestion that specific vitamin supplementation may be effective in the treatment of certain symptoms related to mitochondrial complex 1 deficiency [18]. Several clinical trials also are currently underway to test the effectiveness of therapies in development for mitochondrial disorders [19].

In addition to the *NDUFV2* variant, predicted deleterious homozygous variants were identified in *INO80* (c.G94A; p.D32N) and *PLA2G6* (c.C1696T; pR566W). Aberrations in these genes have been implicated in intellectual disability and neurodegeneration in the first two years of life, respectively. While these variants are of uncertain clinical significance, the diseases associated with the affected genes are consistent with the phenotype of this patient. Our laboratory subsequently learned that this patient had clinical WES at an alternative laboratory and the same homozygous *NDUFV2* variant was identified and the family was counseled accordingly. Because this child passed away in infancy, it is unclear if his clinical course was dictated strictly by the *NDUFV2* variant or if the *INO80* and/or *PLA2G6* variants also contributed.

### Additional rare variants of interest

In addition to the pathogenic or likely pathogenic variants described above, in 74% (39/53) of the patients we identified several homozygous variants that potentially underlie the phenotypes of other patients (Additional file 4). While 29 of the variants we detected are novel and others have not previously been classified clinically, it is likely that at least some of these variants contribute to the clinical findings in these cases, and may eventually be reclassified as pathogenic or likely pathogenic. Notably, 47% (25/53) of the total cohort had multiple rare homozygous variants that were predicted to be deleterious in genes related to the indication for testing, raising the possibility that multiple variants may contribute to the phenotypes in some of these patients, as has been observed in previous clinical sequencing studies [20].

We discuss here one of the compelling cases of rare variant that would be classified clinically as variant of uncertain significance. Details of such variants are provided in Additional file 4, which do not currently meet ACMG guidelines for pathogenic or likely pathogenic classification, but they highlight the potential for much higher diagnostic yields of WES as more information becomes available in the medical literature.

#### KCNAB2

Patient ROH05 was referred to our laboratory with developmental delay and epilepsy. There was no prior report of parental relatedness, but 5.33% of the autosomal genome was found to be homozygous using CMA. No clinically relevant CNVs were present. Using WES, we identified a homozygous missense variant in *KCNAB2* (c.C427T; p.R143W). *KCNAB2* encodes a voltage-gated potassium channel and could be involved in regulating neurotransmitter release and neuronal excitability [21]. Dysfunction of other potassium channels has been shown to be associated with epileptic phenotypes [22]. In a study of individuals with 1p36 deletion syndrome, eight out of nine (89%) patients with hemizygous deletions of *KCNAB2* (which resides in the deleted region) also had epilepsy or epileptiform activity on electroencephalogram [23]. Therefore, haploinsufficiency of *KCNAB2* was suggested as a significant risk factor for epilepsy. In another study, a de novo heterozygous mutation (c.1062dupCA, p.Leu355HisfsTer5) in *KCNAB1* was reported as disease causing for epilepsy in a patient with early infantile epileptic encephalopathy [24]. This mutation in *KCNAB1* resulted in a premature termination codon. *KCNAB1* and *KCNAB2* encode proteins that are members of the cytoplasmic β subunit protein (kvβ) KCNAB family and are paralogous to each other. The β subunits stabilize the α subunits of the protein complex and a decrease in β subunits could reduce K^+^ currents in neurons resulting in fewer functional channels in the membrane. This could result in increased Ca^2+^ entry and neurotransmitter release, causing a hyperexcitable and seizure-prone circuit [23]. Furthermore, deletion of the mouse homolog of *KCNAB2* (*Kcnab2*^*−/−*^) resulted in deficits in associative memory and amygdala hyperexcitability, suggesting that loss of *KCNAB2* contributes to the cognitive and neurological impairments observed in patients with 1p36 deletion syndrome [25]. While patient ROH05 has a homozygous missense variant rather than a heterozygous deletion, if the p.R110W variant has reduced protein function, then it may contribute to the seizure phenotype observed in this patient.

### Heterozygous genetic variants

Almost all the patients in our cohort have phenotypes related to neurodevelopment and we know that neurodevelopmental disorders are a clinically and genetically highly heterogeneous and complex group of disorders. There are multiple genetic factors involved in the etiology of these disorders. Therefore, in addition to the homozygous mutations, we also evaluated heterozygous variants throughout the exome to determine if variation outside the regions of homozygosity might play a role in our patient cohort. In 8/53 (15%) cases, we identified a pathogenic/likely pathogenic variant that explains at least part of the phenotype. Two of these patients (ROH44 and ROH52) also harbor a pathogenic homozygous genetic change relevant to their phenotype, consistent with the idea that multiple genes could be contributing to their clinical symptoms. In our cohort, heterozygous variants relevant to clinical symptoms were more common than homozygous variants found in the ROH. The relevant heterozygous variants are shown in Table 2. Here we discuss genetic diagnosis in one of these cases:

**Table 2:** Details of heterozygous or hemizygous pathogenic/likely pathogenic variants identified in this study

Sample	% ROH	Gene	Chr	Position	HGVS (nucleotide)	HGVS (protein)	Variation type	ExAC Allele Count/Total Allele No.	Classification	Associated Disorders
ROH07	10	*PNPLA4*	X	7,870,101	c.559C > T	p.R187*	Stop-gain	2/87731	Likely pathogenic	Mitochondrial respiratory chain complex deficiencies
ROH21	5.89	*CADM1*	11	115,088,681	c.752A > C	p.Y251S	Missense	42/121050	Likely pathogenic	Susceptibility to ASD
ROH23	3.6	*HBB*	11	5,248,155	c.92 + 5G > C	–	Splice region	87/121280	Likely pathogenic	Beta thalassaemia
ROH23	3.6	*HBB*	11	5,248,224	c.27dupG	p.Ser10Valfs*14	Frameshift	34/121344	Likely pathogenic	Beta thalassaemia
ROH34	5.3	*SOS1*	2	39,278,394	c.755 T > C	p.I252T	Missense	9/121342	Pathogenic	Noonan syndrome
ROH37	6.13	*SFTPC*	8	22,020,159	c.115G > A	p.V39M	Missense	4/120740	Likely pathogenic	Interstitial lung disease
ROH44	24.55	*OTC*	X	38,226,630	c.164A > G	p.Y55C	Missense	0/121412	Likely pathogenic	Ornithine transcarbamylase deficiency
ROH45	15.8	*PNPLA4*	X	7,870,101	c.559C > T	p.R187*	Stop-gain	2/87731	Likely pathogenic	Mitochondrial respiratory chain complex deficiencies
ROH52	18.1	*ASMT*	X	1,748,834	c.562 + 2 T > C	–	Splice donor	168/121412	Pathogenic	Susceptibility to ASD

#### *OTC*-likely pathogenic variant

In the patient ROH44 where we identified a homozygous variant in *TPP1* gene, we identified an X-linked likely pathogenic hemizygous variant in the *OTC* gene (c.164A > G, p.Y55C). The gene *OTC* encodes a mitochondrial matrix enzyme, ornithine carbamoyltransferase. Genetic variants in this gene including missense, nonsense and frameshift mutations as well as deletions and duplications in this gene results in ornithine transcarbamylase deficiency, which causes hyperammonemia [26–28]. Another variant at this position p.Y55D has been reported to be associated with late-onset hyperammonemia in a male patient [29]. Functional studies were performed and the variant p.Y55D was shown to be disease-associated. It was shown that the genetic change at position 55 is likely to destabilize the mutant OTC as the tyrosine residue is in the helix 1 of the OTC subunit which is conserved in humans and rodents [29]. The typical neuropsychological complications for individuals with OTC deficiency include developmental delay, learning disabilities, intellectual disability, attention deficit hyperactivity disorder (ADHD), executive function deficits and seizures. Biochemical tests in the family can be performed to confirm the diagnosis in this case. Although these data were generated as part of a research study, the molecular diagnoses we obtained would prompt clinical management changes in these individuals (Table 3).

**Table 3:** Guideline-recommended clinical management changes associated with pathogenic or likely pathogenic mutations

Sample	Gene	Clinical management changes/notifications based on molecular diagnosis
ROH04	*TYR*	Ophthalmologic evaluations at least annually along with routine skin screening for pre-cancerous lesions due to increased risk for melanoma. Appropriate preparation for sun exposure (sunscreen, glasses, hats, etc). Ongoing clinical trials to evaluate proposed treatment response on cultured melanocytes.
ROH22	*PCCB*	Management in a metabolic clinic including dietary guidance and supplementation. Screen for cardiomyopathy and cardiac dysfunction. Intravenous provision of glucose and lipids when undergoing acute infection, dehydration, or vomiting. Avoidance of prolonged fasting, excess protein intake, medications that prolong QT interval and neuroleptic antiemetics.
ROH26	*SLC25A15*	Management in metabolic clinic including dietary guidance (low-protein diet, citrulline supplementation) and use of ammonia scavengers. Avoidance of liver transplantation, prolonged fasting, and valproic acid.
ROH31	*NDUFV2*	Consideration of mitochondrial treatments/supplementation including riboflavin and screening for hypertrophic cardiomyopathy.
ROH44	*TPP1*	Seizure medications that may be contraindicated include carbamazepine, phenytoin, lamotrigine. Ongoing clinical trials for recombinant human enzyme therapy, as well as ongoing natural history, genotype-phenotype, and other studies.
ROH52	*GJB2*	Multidisciplinary management (ex: geneticist, otolaryngologist, deaf educator, etc.) and consideration of cochlear implantation. Avoidance of noise exposure.
ROH23	*HBB*	Specific treatments (e.g. transfusions, managing related issues such as potential iron overload), can be effective. Mutations may also result in different types of hematologic disease (e.g. Hemolytic anemia), and genetic diagnosis may aid early recognition and treatment (e.g. with RBC transfusion), as well as avoid unnecessary treatments (e.g. splenectomy in Heinz body anemia).
ROH44	*OTC*	Long-term dietary measures (e.g. decreasing the nitrogen load with low protein diet, use of nitrogen scavengers, and administration of arginine/citrulline) may be beneficial. Certain agents (e.g. valproate, systemic corticosteroids, as well as triggers such as fasting) should be avoided due to the potential of adverse events.

## Discussion

Homozygosity in the genome raises the possibility of conditions that exhibit autosomal recessive inheritance; therefore, clinical laboratories typically proceed with targeted sequencing by selecting candidate genes within the homozygous regions based on a patient’s phenotype. This approach can be used in cases where the patient’s clinical presentation strongly suggests a specific genetic disorder and the number of genes consistent with the phenotype is limited. However, in many cases CMA may not detect all the regions of homozygosity and the complex phenotype may not allow for identification of strong candidate genes. Investigating the ROH detected by CMA to identify genes that exhibit an autosomal recessive inheritance pattern followed by specific sequencing of those genes is a cumbersome and inefficient process and has the possibility to miss causative mutations. It is important to note that there is a variable size threshold for ROH in clinical settings with some labs reporting only regions larger than 10 Mbp [30]. Most clinical labs, if not all, would not report ROH below 3 Mbp due to the limitations of the CMA platforms. Our laboratory uses a minimum of 3 Mb to report ROH. Importantly, the targeted approach becomes more unreliable as the phenotype becomes less specific or more complex and as the homozygosity levels become higher. Currently, there are several tools to select candidate genes for targeted sequencing. However, irrespective of the tool(s) we use, a separate gene panel, with a separate list of potential candidate genes, would need to be designed for every patient based on their ROH and their clinical features. With the low cost of WES, it is much more effective to perform WES rather than gene panels for targeted sequencing. In cases where we cannot identify the genetic cause using an autosomal recessive model, WES allows us to search immediately for X-linked variants as well as heterozygous variants with an autosomal dominant inheritance pattern. This would not be possible using the gene panel approach. Furthermore, as new disease genes are continuously being discovered, gene panels may become outdated rather quickly and updating them for targeted sequencing is a costly and time-consuming process. In contrast, WES allows for re-interpretation as new gene-disease associations are determined. Our data suggest that follow up testing using WES is a more effective approach as some of the pathogenic/likely pathogenic variants identified in this study did not lie within the ROH.

From a clinical perspective, these data furnish genetic and other healthcare professionals with additional critical and objective information to communicate to families whose child has ROH observed on SNP-based CMA. Moreover, as reimbursement for genetic testing is limited by specific health plan policies and clinical guidelines, our results support a recommendation for the use of WES following detection of ROH from SNP-based CMA to gain a better understanding of the genetic cause of a patient’s condition. We highlighted some of the important management changes potentially enabled by our results in a number of these patients, and the less established findings are likely to lead to such changes in the future.

In our cohort, the diagnostic yield of exome sequencing technology is 22% (12/53 cases had at least one pathogenic/likely pathogenic mutation) when we evaluate homozygous, heterozygous and X-linked variants across the whole exome. It is important to note that many of the causative mutations lie outside the regions of homozygosity, which is an important point to consider when a targeted or a whole exome sequencing test is being considered. In 41/53 (77%) of our patients, no diagnostic variant was identified. It is possible that in some of these patients, the causative mutation(s) lie in an exon not well covered by the exome capture methodology used, or in a non-coding/regulatory region, which are not targeted by WES. However in several of these patients, we identified homozygous rare variants that are classified as variants of uncertain significance (listed in Additional file 4). As medical literature becomes more complete some of these variants may be re-classified as pathogenic or likely pathogenic, resulting in an increase in diagnostic yield. It is important to recognize that even in cases with a homozygous pathogenic variant in a gene that clearly matches a patient’s symptoms, additional variants uncovered by WES may have additional effects on symptomology, and these may contribute to further, more precise medical management. De novo mutations appear to be enriched in neurodevelopmental cohorts. Since we did not have parental sequence data, analysis of de novo variant status could not be performed, and this likely would have increased the diagnostic yield. A recent study showed that of 75% of children who undergo whole-exome sequencing receive no diagnosis, but reanalysis of their exomes using updated software and literature may yield a diagnosis of 10% of these patients [31]. Future analyses of these non-diagnostic exomes will likely increase the proportion of patients who get an answer from exome sequencing in this cohort.

Some of the patients in which we have identified pathogenic variants, for example *TYR* for albinism, have additional phenotypes like seizures which are not explained by the observed genetic variant. There may be additional variants in other genes that explain some of the other phenotypes of these patients. Another point to consider is that about 30% (16/53) of patients in this cohort have homozygosity encompassing only 1.4–3% of their genomes. Some of the regions of homozygosity in these patients may reflect the presence of common haplotypes in a population. Because this study is focused on clinical samples where sequencing has only been performed on patients with neurodevelopmental phenotypes, we cannot estimate the burden of similar genetic variants in controls with similar number or size of ROH.

Over the past few years, several studies have shown the diagnostic utility of exome sequencing in clinical diagnostic laboratories. One of the studies by Baylor College of Medicine was carried out on 250 consecutive probands and approximately 80% were children with phenotypes related to neurologic conditions [32]. Prior genetic testing consisting of CMA, metabolic screening or DNA sequencing identified 15 positive cases. Using WES, the overall rate of a positive molecular diagnosis was 25% (62/250). In some follow-up study of 2000 patients, the same diagnostic yield of 25% was observed [20]. Another study discussed that WES is most cost-effective early in the diagnostic trajectory, but after chromosomal microarray analysis has been performed [33]. WES was likely to have the highest diagnostic yield for those patients with genetically heterogeneous disorders or features overlapping several conditions and they would be best served by early referral to Clinical genetics with WES applied after chromosomal microarray analysis but before other more extensive diagnostic processes. To our knowledge, there have not been many studies discussing the clinical utility of whole exome sequencing in individuals with ROH. Our study illustrates the clinical utility of WES in these individuals and suggest that many of these individuals not only have causative variants within the regions of ROH, but also heterozygous/hemizygous variants outside these regions that may help explain the phenotype of these patients. Therefore, the appropriate testing should be whole exome sequencing following chromosomal microarray analysis rather than the traditional targeted panel test for individuals with ROH.

## Conclusion

WES identified numerous variants that are relevant or potentially relevant to the phenotype of patients with ROH identified by SNP-based CMA. In (12/53) cases, we identified at least one pathogenic or likely pathogenic variant that explains at least part of the indicated phenotype of the patients. We identified an equal number of pathogenic/likely pathogenic variants outside the ROH, suggesting that WES is more appropriate than targeted sequencing in these patients. Importantly, the variants we identified provide definitive diagnoses that offer valuable information for future medical care as well as recurrence risk and family planning options. As similar cases are reported and as functional data become available, other variants identified in this study are likely to be re-classified as pathogenic or likely pathogenic. Therefore, this study demonstrates that whole exome sequencing, rather than targeted sequencing, has clinical utility in individuals with ROH identified on CMA, and should be considered for inclusion in clinical guidelines for the staged evaluation of individuals with neurodevelopmental disorders.

## Additional files

Additional file 1: A detailed workflow showing the variants discovery and prioritization for the individuals with ROH. (DOCX 27 kb)

Additional file 2: Clinical Phenotype information for the 53 ROH patients along with CMA findings. (XLSX 17 kb)

Additional file 3: A scatter plot showing correlation of percent of homozygosity with the number of homozygous likely gene disrupting variants predicted to be deleterious. At this stage, no frequency filter was applied. (DOCX 124 kb)

Additional file 4: Details of rare homozygous variants discovered in 74% (39/53) of the ROH cases that has variants relevant or potentially relevant to the phenotype of the patient. (XLSX 32 kb)

## Abbreviations

ADHD: Attention-deficit hyperactivity disorder
ACMG: American College of Medical Genetics and Genomics
ASD: Autism spectrum disorder
CMA: Chromosomal microarray analysis
DD: Developmental delay
HHH: Hyperornithinemia-hyperammonemia-homocitrullinuria
ID: Intellectual disability
Kbp: Kilo base pairs
Mb: Megabases
NDDs: Neurodevelopmental disorders
ROH: Regions of homozygosity
SNP: Single nucleotide polymorphism
VEP: Variant effect predictor
VOUS: Variants of uncertain significance
WES: Whole Exome Sequencing
