# Supplementary material for: Clinical utility of exome sequencing in individuals with large homozygous regions detected by chromosomal microarray analysis
Source: BMC Med Genet. 2018 Mar 20;19:46. doi: 10.1186/s12881-018-0555-3 (PMC5859484; doi:10.1186/s12881-018-0555-3)
Supplement: Supplementary file 1 — A detailed workflow showing the variants discovery and prioritization for the individuals with ROH. (DOCX 27 kb) [file 12881_2018_555_MOESM1_ESM.docx]

**Additional File 1.**
