# Supplementary material for: Clinical utility of exome sequencing in individuals with large homozygous regions detected by chromosomal microarray analysis
Source: BMC Med Genet. 2018 Mar 20;19:46. doi: 10.1186/s12881-018-0555-3 (PMC5859484; doi:10.1186/s12881-018-0555-3)
Supplement: Supplementary file 3 — A scatter plot showing correlation of percent of homozygosity with the number of homozygous likely gene disrupting variants predicted to be deleterious. At this stage, no frequency filter was applied. (DOCX 124 kb) [file 12881_2018_555_MOESM3_ESM.docx]

**Additional File 3.**
